# High proportions of multidrug-resistant *Klebsiella pneumoniae* isolates in community-acquired infections, Brazil

**DOI:** 10.1038/s41598-025-92549-w

**Published:** 2025-03-20

**Authors:** Adriano de Souza Santos Monteiro, Márcio de Oliveira Silva, Vívian Santos Galvão, Adriele Pinheiro Bomfim, Lorena Galvão de Araújo, Camila Maria Piñeiro Silva, Maria Goreth Barberino, Edilane Lins Gouveia, Soraia Machado Cordeiro, Joice Neves Reis

**Affiliations:** 1 Postgraduate Course in Biotechnology in Health and Investigative Medicine, Gonçalo Moniz Institute, Oswaldo Cruz Foundation, Salvador, BA Brazil; 2 São Rafael Hospital, Salvador, BA Brazil; 3 Postgraduate Program in Pharmacy, Faculty of Pharmacy, Federal University of Bahia, Salvador, BA Brazil; 4 Bahia Hospital, Salvador, BA Brazil; 5 Faculty of Pharmacy, Federal University of Bahia, Ondina, Salvador, 40170-115 BA Brazil

**Keywords:** *Klebsiella pneumoniae*, Urinary tract infections, Bloodstream infections, Community-acquired, Antimicrobial resistance, High-risk clones, Microbiology, Antimicrobial resistance

## Abstract

*Klebsiella pneumoniae* is one of the leading causes of bloodstream (BSI) and urinary tract infections (UTI), but limited data is available regarding community-acquired (CA) infections. This study characterized the clinical aspects of CA-BSI and CA-UTI caused by *K. pneumoniae* and the molecular features of isolates, including their resistance profiles. Sixty-five isolates (CA-BSI, *n* = 24; CA-UTI, *n* = 41) underwent antimicrobial susceptibility testing, β-lactamase and virulence gene assessment, capsular genotyping, and molecular typing. Older age, male gender, and comorbidities, particularly kidney disease, were significantly associated with CA-BSI. The MDR and carbapenem resistance rates for *K. pneumoniae* from CA infections were 24.6% and 4.6%, respectively. CA-BSI isolates were more antibiotic-resistant and had a higher proportion of ESBL-producing (37.5% *versus* 9.8%) and MDR isolates (45.8% *versus* 12.2%) than CA-UTI. The *bla*_CTX−M−like_ or *bla*_KPC−like_ genes was found in all ESBL-producing isolates, while *bla*_KPC−like_ and *bla*_NDM−like_ were detected exclusively in CA-BSI strains. The isolates’ virulence profiles were similar between the groups, although one CA-BSI and two CA-UTI isolates presented hypervirulence biomarkers. A high clonal diversity was observed, with a majority of MDR (81.3%) (ST11, ST15, ST101, ST258, ST307, and ST6852) and hypervirulent (2/3) (ST23 and ST65) isolates being high-risk pandemic clones in humans. Our data highlight the high prevalence of MDR *K. pneumoniae* in CA infections in Brazil, with CA-BSI showing significant differences in resistance profiles compared to CA-UTI.

**Supplementary Information:**

The online version contains supplementary material available at 10.1038/s41598-025-92549-w.

## Introduction

The bloodstream and urinary tract are the sites most infected by *Klebsiella pneumoniae*^1,2^, and infections at these sites are significant causes of morbidity and mortality globally^1,3^. *K. pneumoniae* is also recognized as an important cause of community-acquired (CA) infections, and the emergence of hypervirulent *K. pneumoniae* (hv*Kp*) has stood out for its high capacity to cause invasive and severe infections in recent decades^2^. Nowadays, *K. pneumoniae* infections are difficult to treat due to the high level of antibiotic resistance^4,5^, posing a significant threat to public health.

Antimicrobial resistance is no longer restricted to hospitals, and multidrug-resistant (MDR) *K. pneumoniae* infections are now reported in CA infections^6–9^. The production of β-lactamase enzymes is the main resistance mechanism in *K. pneumoniae*, and infections due to extended-spectrum beta-lactamase (ESBL)-and carbapenemase-producing *K. pneumoniae* have dramatically increased worldwide^10^.

Several hospital or healthcare-acquired infections caused by *K. pneumoniae* have been described in Brazil and worldwide, including bloodstream infections (BSI) and urinary tract infections (UTI)^11–14^. However, studies that aim to characterize *K. pneumoniae* in specific cases of CA-BSI and CA-UTI often focus on the clinical characteristics of the patients, leaving aside the analysis of the virulence or clonal profile of the isolates^6–9,15^. Furthermore, as observed in previous Brazilian studies, many CA infection studies are restricted to outpatients^5,16^. In this context, we aimed to determine the proportions of CA-BSI and CA-UTI cases caused by *K. pneumoniae* and characterize the clinical aspects of these infections. Additionally, we aimed to conduct a molecular characterization of the isolates and assess their resistance profiles.

## Materials and methods

Ethics clearance for this study was obtained from the Research Ethics Committee of the Federal University of Bahia (UFBA) under authorization numbers 2.170.080/2017 and 2.012.382, and the Medical Board and Ethics Committee of the São Rafael Hospital (2.415.920) and Hospital da Bahia (2.327.545). Both hospitals’ Ethics Committees approved the study and granted a waiver for informed consent for the retrospective review of patient medical records. For the patients with UTI, informed consent was obtained from all participants. All study protocols complied with the ethical principles outlined in the Declaration of Helsinki and adhered to Resolution 466/2012 of the Brazilian National Health Council for research involving human subjects.

### Study design and population

This study included samples of patients with CA-BSI obtained from a cross-sectional study conducted at two private tertiary hospitals in Salvador, Brazil, from March 2015 to May 2019. Samples from CA-UTI patients were collected during a surveillance study at the Clinical Analysis Laboratory of the School of Pharmacy, Federal University of Bahia, also in Salvador, Brazil, from April 2019 to July 2023. The population with both CA-BSI and CA-UTI consisted of patients without age or sex restrictions, with positive blood or urine cultures for *K. pneumoniae*. Clinical and demographic data of the patients and microbiological data of the isolates from each infection group were analyzed and compared.

### Data collection and definitions

Clinical and demographic data from patients with BSI were collected through medical chart reviews, whereas data for patients with UTI were obtained through questionnaire interviews. Patient comorbidities were analyzed individually and combined using the Charlson comorbidity index^17^. The severity of BSI was measured based on the Pitt bacteremia score^18^, and sepsis was defined according to the Sepsis-2 criteria^19^.

CA infections were defined as cases in which patients did not meet the hospital-acquired infection criteria (occurred 48 h after hospital admission) or healthcare-associated infection criteria^20^: (i) received intravenous therapy at home or in an outpatient clinic within 30 days before the infection; (ii) received renal dialysis in a hospital or hemodialysis clinic in the 30 days before the infection; (iii) received wound care or specialized nursing care in the 30 days before the infection; (iv) surgery or hospitalization in the 90 days before the infection; (v) lived in a nursing home or long-term care institution in the 12 months before the infection; (vi) subjected to invasive urinary procedures or use of long-term urinary catheters in the 30 days before the infection. CA-BSI was also defined as a positive blood culture occurring ≤ 48 h after hospital admission.

Primary BSI was determined when no source of extravascular infection was identified. In contrast, secondary BSI was defined when the microorganism isolated from a blood culture was related to an infection at another site. Isolates resistant to at least three different categories of antibiotics were considered multidrug-resistant^21,22^.

### Bacterial identification and antimicrobial susceptibility testing

The blood cultures were performed on BacT/Alert® (bioMérieux), and *K. pneumoniae* was identified by Vitek® 2 (BioMérieux ™) or MALDI-TOF (Matrix Assisted Laser Desorption Ionization - Time of Flight Mass Spectrometry) (BioMérieux ™). For urine cultures, a positive result was defined as ≥ 100,000 CFU/mL, and *K. pneumoniae* was identified using standard biochemical tests. Antimicrobial susceptibility testing was conducted using Vitek® 2 (BioMérieux™) for CA-BSI isolates and the disk diffusion method for CA-UTI isolates, following the Clinical and Laboratory Standards Institute (CLSI)^23^ guidelines. Additionally, ESBL production was verified using the CLSI phenotypic confirmatory disc test. Colistin resistance was initially screened using the drop test^24^ and minimum inhibitory concentration values for positive isolates were determined by broth microdilution. *Escherichia coli* ATCC 25922 was used as quality control.

### Detection of β-lactamase and virulence genes

Genomic DNA was extracted using the QIAamp DNA Mini Kit (Qiagen) and subjected to PCR reactions to identify the β-lactamase genes *bla*_SHV_, *bla*_TEM_, *bla*_OXA−1−like_, *bla*_CTX−M_, *bla*_GES_, *bla*_OXA−48−like_, *bla*_IMP_, *bla*_VIM_, *bla*_KPC_, and *bla*_NDM_^25,26^. PCR reactions were also conducted to identify genes associated with the capsule (_*p*_*rmpA*, _*p*_*rmpA2*, *wabG*, and *uge*), siderophores (*entB*, *ybtS*, *iucA*, and *iroB*), fimbriae (*fimH* and *mrkD*), iron uptake system (*kfu*), ability to metabolize allantoin (*allS*), urease production (*ureA*), serum resistance (*traT*), toxin production (*hlyA* and *cnf*-1), and putative metabolite transporter (*peg-344*, located on the virulence plasmid)^27–30^. The PCR products were subsequently analyzed by agarose gel electrophoresis as described by the mentioned authors. Primers and product sizes are listed in Table S1 in the supplemental material.

The definition of hv*Kp* was the presence of some combination of *peg-344*,* iroB*,* iucA*, _*p*_*rmpA*, or _*p*_*rmpA2* genes^29^. The remaining isolates were defined as classical *K. pneumoniae* (c*Kp*).

### Capsular typing

Capsular variation was analyzed based on the allelic diversity of the *wzi* gene, which was amplified and sequenced using the Sanger method, as described by Brisse et al.^31^. Allele analysis and determination of the putative K-type (or KL-type), when possible, were conducted according to the scheme established in the Pasteur Institute *K. pneumoniae* BIGSdb database (https://bigsdb.pasteur.fr/klebsiella/). New *wzi* variants identified were submitted to the Pasteur Institute to obtain new alleles. Serotypes K1 and K2 were confirmed through PCR^27^ and Sanger sequencing.

### Molecular typing

The isolates were subjected to pulsed-field gel electrophoresis (PFGE) analysis using *Xba*I-digested DNA with a CHEF-DRIII apparatus (Bio-Rad Laboratories, Inc., Hercules, CA, USA). Electrophoresis conditions were voltage 6 V/cm, running time of 18 h, temperature of 14 °C, and pulse times ranging from 1 to 40 s. PFGE Lambda Ladder (New England Biolabs, Germany) was used as a base pair size marker. Genetic similarity was evaluated using GelCompar II® software (Applied Maths, Kortrijk, Belgium) with a DICE coefficient (1.5% tolerance).

The isolates were further analyzed through multilocus sequence typing (MLST) using PCR and sequencing of seven housekeeping genes (*gapA*, *infB*, *mdh*, *pgi*, *phoE*, *rpoB*, and *tonB*), following the protocol provided by Pasteur Institute (https://bigsdb.pasteur.fr/klebsiella/). The MLST data analysis was performed using the goeBURST algorithm implemented in the PHYLOViZ program (http://www.phyloviz.net/goeburst/). Clusters were defined as groups of sequence types (STs) sharing six identical loci, including the founder ST and its single-locus variants (SLVs).

### Statistical analysis

Fisher’s exact test was used to analyze categorical variables, while the Mann-Whitney test was applied to continuous variables. Values of *p* < 0.05 (95% confidence interval) were considered statistically significant. Statistical analysis was performed in GraphPad Prism v. 8.0.1 for Windows (GraphPad Software, La Jolla, CA, USA).

## Results

### Demographic and comorbidity characteristics of patients with CA-BSI and CA-UTI caused by *K. pneumoniae*

This study included 65 patients with CA infections. Among the 278 episodes of *K. pneumoniae* BSI identified during the study period, 24 (8.6%) were CA cases. The remaining 41 cases were CA-UTI patients, randomly selected from a total of 95 *K. pneumoniae* UTI cases occurring in the study period. Patients with CA-BSI were significantly older than those with CA-UTI (median age 76 *versus* 47; *p* < 0.01), and the prevalence of male patients was higher among patients with CA-BSI (66.7% *versus* 12.2%; *p* < 0.01). In terms of comorbidities addressed by the Charlson score, patients with CA-BSI had more comorbidities (*p* < 0.01), especially kidney disease (*p* < 0.01), as shown in Table 1.

**Table 1:** Demographic and clinical characteristics of patients with community-acquired bloodstream infections (*n* = 24) and community-acquired urinary tract infections (*n* = 41) caused by *Klebsiella pneumoniae*.

Characteristics	CA-BSI	CA-UTI	*p* ^a^
	24 (%)	41 (%)	
**Demographic data**			
Female	8 (33.3)	36 (87.8)	**< 0.01**
Male	16 (66.7)	5 (12.2)	
Age (Years), median (1 qt–3 qt)^b^	76 (62–83)	47 (24–66)	**< 0.01**
0–15	1 (4.2)	3 (7.3)	> 0.99
16–30	1 (4.2)	10 (24.4)	**0.04**
31–59	3 (12.5)	14 (34.1)	0.08
60–80	10 (41.7)	12 (29.3)	0.42
≥ 81	9 (37.5)	2 (4.9)	**< 0.01**
**Comorbidities**			
Charlson comorbidity index (median [1 qt-3 qt]) ^b^	3 (1–4)	0 (0–2)	**< 0.01**
Charlson comorbidity index ≥ 5	5 (20.8)	1 (2.4)	**0.02**
Any	15 (62.5)	7 (17.1)	**< 0.01**
Diabetes	6 (25.0)	6 (14.6)	0.33
Liver disease	2 (8.3)	1 (2.4)	0.55
Rheumatoid arthritis	–	1 (2.4)	> 0.99
Kidney disease	5 (20.8)	–	**< 0.01**
Dementia	3 (12.5)	–	**0.046**
History of malignancy	2 (8.3)	–	0.13
Others	*	–	0.37

BSI, bloodstream infection; CA, community-acquired; qt, quartile; UTI, urinary tract infection. ^a^ Boldface indicates significance; ^b^ Mann-Whitney U test; other comparisons were performed using Fisher’s exact test. * Congestive heart failure (4.2%; 1/24), peripheral vascular disease (4.2%; 1/24), chronic obstructive pulmonary disease (4.2%; 1/24), metastatic disease (4.2%; 1/24), HIV/AIDS (4.2%; 1/24), cerebrovascular disease (4.2%; 1/24), peptic ulcer (4.2%; 1/24), hemiplegia or paraplegia (4.2%; 1/24).

### Clinical outcomes of CA-BSI patients

Concerning the characteristics of CA-BSI episodes, most infections had a secondary source (66.7%, 16/24), with the urinary tract (50.0%, 8/16) being the most frequent site of origin, followed by the respiratory tract and intra-abdominal (18.8%, 3/16 each). Among patients with CA-BSI, 29.2% (7/24) exhibited severe bacteremia based on the Pitt score, and 50.0% (12/24) experienced systemic complications due to bacteremia, such as severe sepsis or septic shock (Table 2). Notably, 25.0% (6/24) of CA-BSI patients died, and among those, 50.0% (3/6) had kidney disease.

**Table 2:** Clinical characteristics of patients and source of the community-acquired bloodstream infections caused by *Klebsiella pneumoniae* (*n* = 24).

Type of CA-BSI	*n* = 24	%
Pitt bacteremia score (median [1 qt–3 qt])	2	0–5
Pitt score ≥ 4	7	29.2
Primary source	8	33.3
Secondary source	16	66.7
Urinary tract	8	50.0
Respiratory tract	3	18.8
Intra-abdominal	3	18.8
Gastrointestinal tract	1	6.3
Osteomyelitis	1	6.3
Severe sepsis or septic shock^a^	12	50.0
Crude mortality	6	25.0

BSI, bloodstream infection; CA, community-acquired. ^a^ Severe sepsis, characterized by organ dysfunction, could progress to septic shock, defined as persistent hypotension induced by sepsis despite adequate fluid resuscitation.

### Antimicrobial resistance between CA-BSI and CA-UTI *K. pneumoniae* isolates

**Fig. 1:**
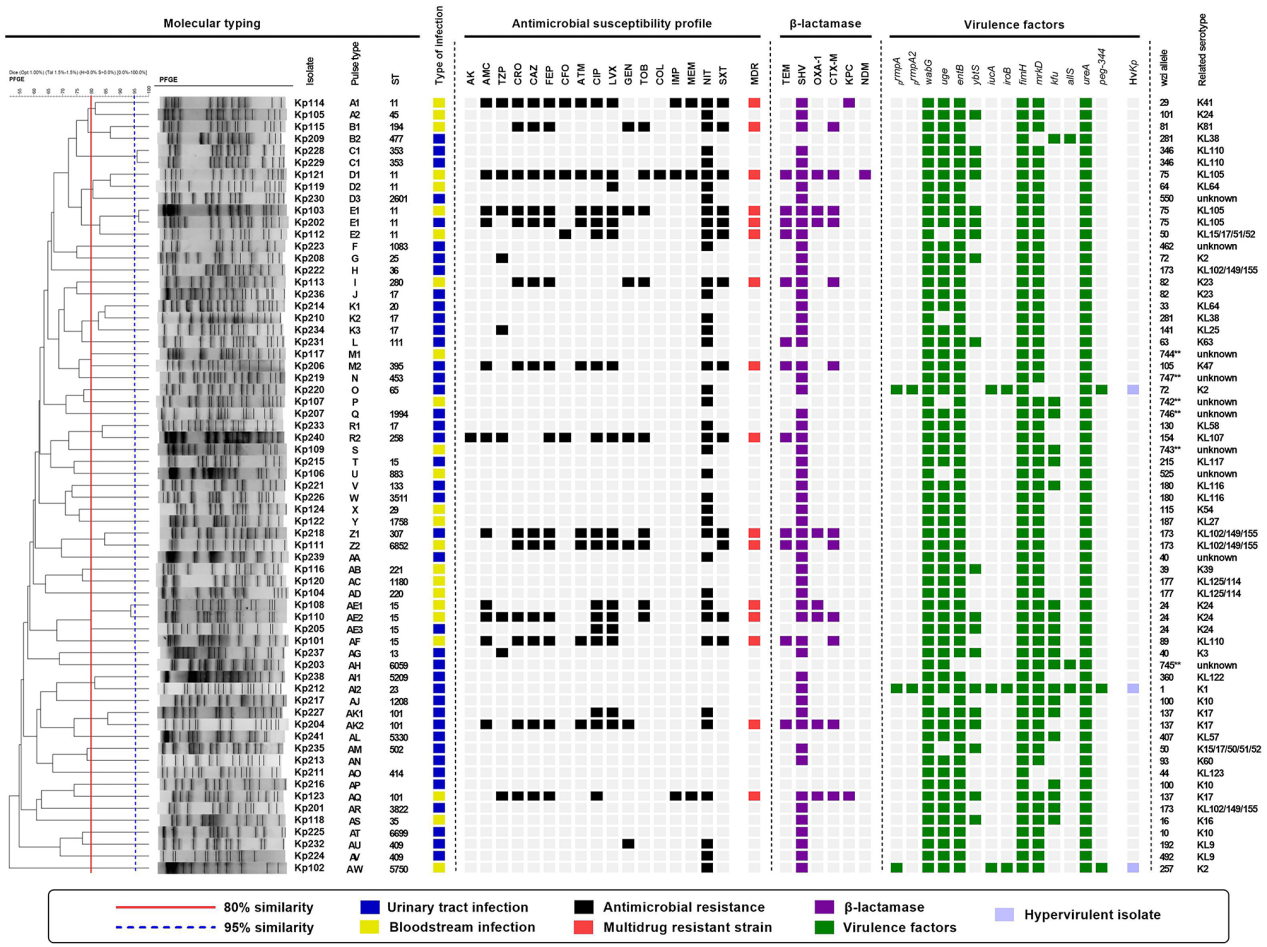
Molecular profile of *Klebsiella pneumoniae* from community-acquired bloodstream infections and community-acquired urinary tract infections (*n* = 65). AK, amikacin; AMC, amoxicillin-clavulanic acid; ATM, aztreonam; CAZ, ceftazidime; CFO, cefoxitin; CIP, Ciprofloxacin; COL, colistin; CRO, ceftriaxone; FEP, cefepime; GEN, gentamicin; hv*Kp*, hypervirulent *Klebsiella pneumoniae*; IMP, imipenem; LVX, levofloxacin; MDR, multidrug-resistant; MEM, meropenem; NIT, nitrofurantoin; ST, sequence typing; SXT, trimethoprim-sulfamethoxazole; TOB, tobramycin; TZP, piperacillin-tazobactam. *New ST; **New *wzi* alleles.

**Fig. 2:**
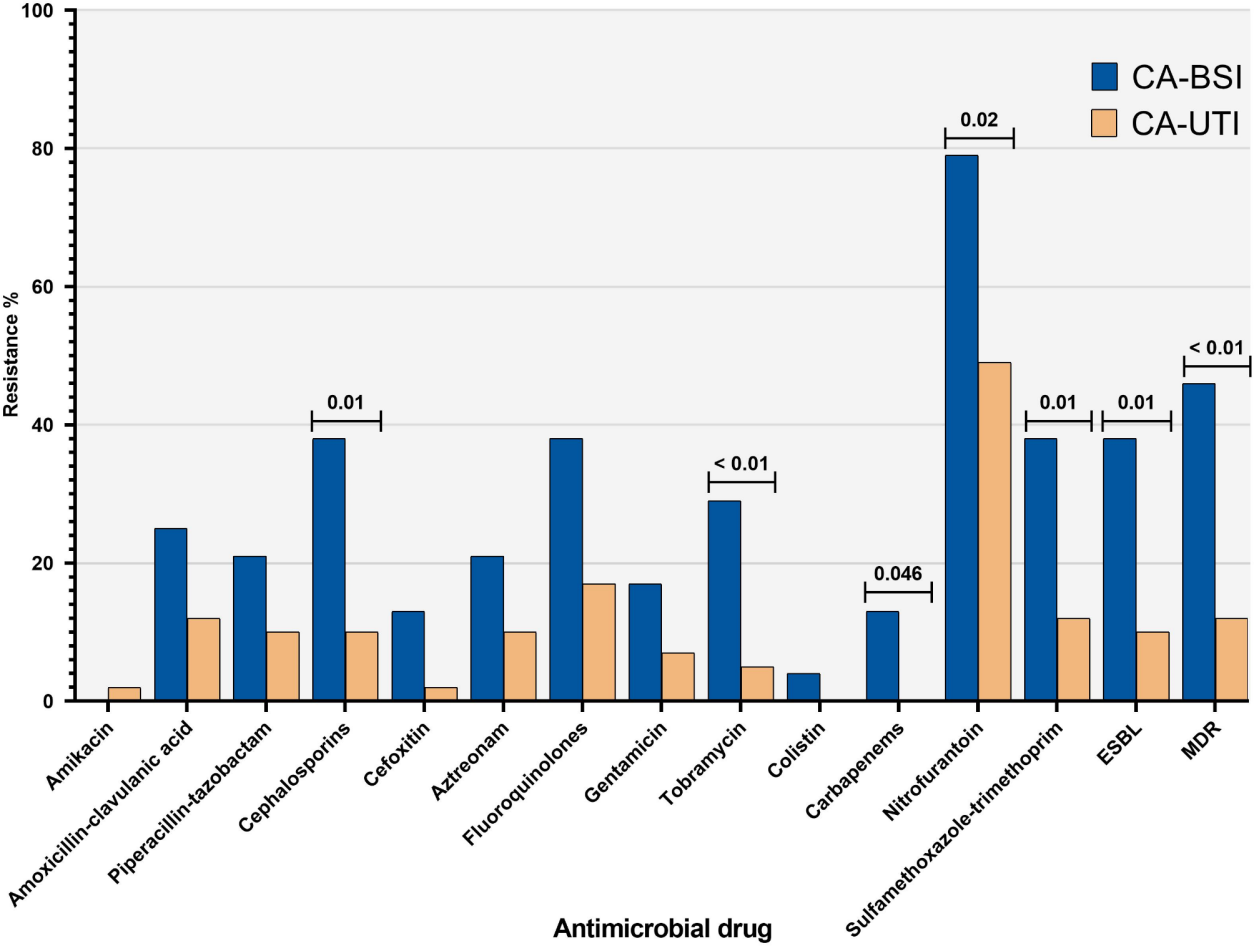
Frequency of antimicrobial resistance of *Klebsiella pneumoniae* from community-acquired bloodstream infections (*n* = 24) and community-acquired urinary tract infections (*n* = 41). BSI, bloodstream infection; CA, community-acquired; ESBL, extended-spectrum β-lactamase; MDR, multidrug resistance; UTI, urinary tract infection.

The MDR and carbapenem resistance rates for all *K. pneumoniae* from CA infections in this study were 24.6% (16/65) and 4.6% (3/65), respectively (Fig. 1). When we compared the antimicrobial susceptibility test results among isolates from the patient groups, we observed that *K. pneumoniae* from CA-BSI were more resistant than those from CA-UTI, particularly to cephalosporins (*p* = 0.01), tobramycin (*p* < 0.01), carbapenems (*p* = 0.046), nitrofurantoin (*p* = 0.02), and sulfamethoxazole-trimethoprim (*p* = 0.01). CA-BSI *K. pneumoniae* isolates also exhibited a significantly higher rate of ESBL production confirmed by the phenotypic disc test (9/24; 37.5% *versus* 4/41; 9.8%; *p* = 0.01) and MDR (11/24; 45.8% *versus* 5/41; 12.2%; *p* < 0.01) when compared with CA-UTI isolates. Notably, resistance to carbapenems was only found in cases of CA-BSI (Fig. 2).

**Fig. 3:**
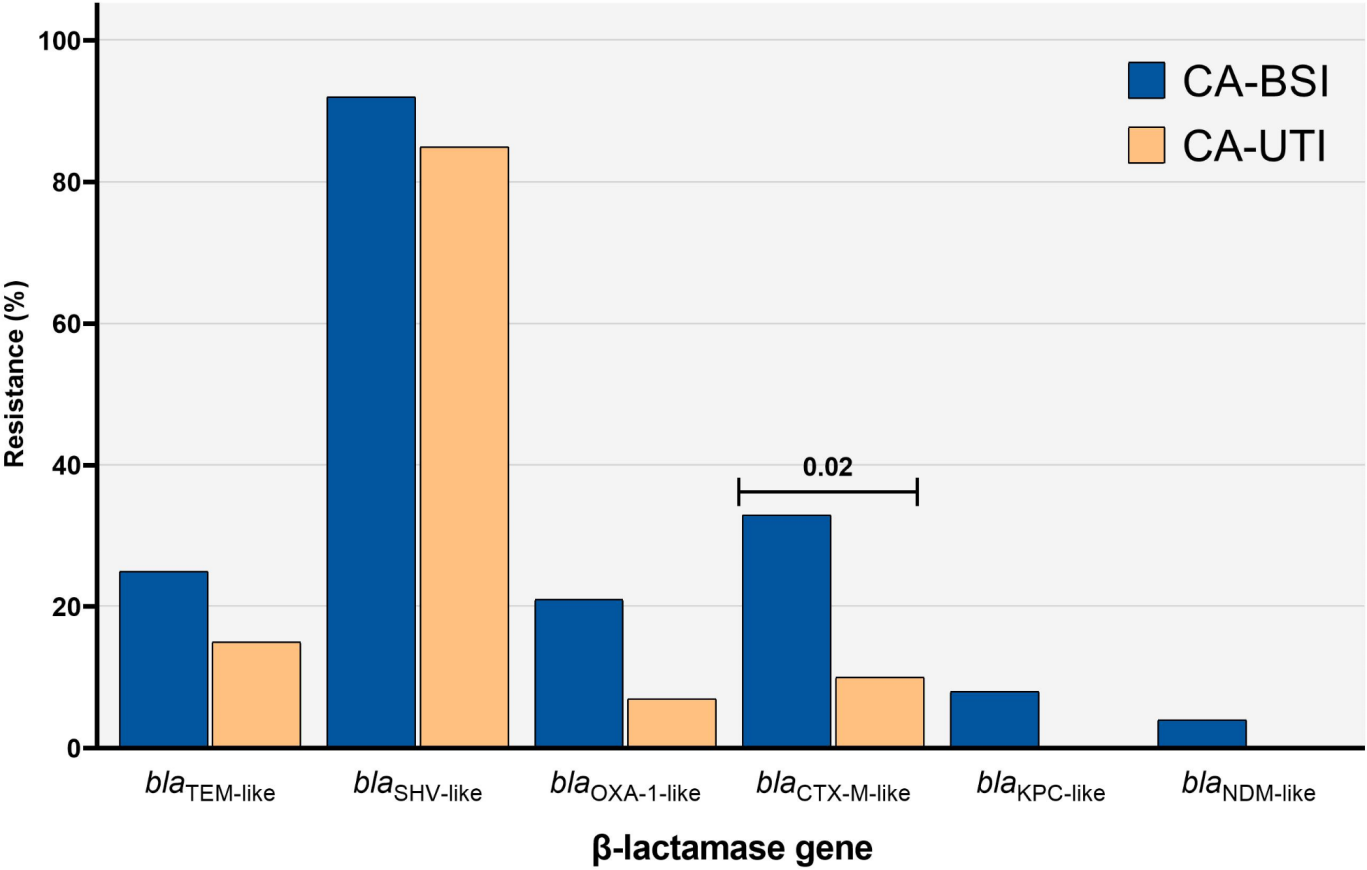
Frequency of β-lactamases genes in *Klebsiella pneumoniae* from community-acquired bloodstream infections (*n* = 24) and community-acquired urinary tract infections (*n* = 41). BSI, bloodstream infection; CA, community-acquired; UTI, urinary tract infection.

We further analyzed the β-lactamase gene content of *K. pneumoniae* isolates and identified 11 distinct patterns (A to K) (Table 3). The frequency of β-lactamase genes was higher among CA-BSI isolates than CA-UTI (Fig. 3), supporting the phenotypic findings of the antimicrobial susceptibility test. All ESBL-producing *K. pneumoniae* confirmed by the phenotypic disc test possessed the *bla*_CTX−M−1_, *bla*_CTX−M−9_ or *bla*_KPC−like_ genes, predominantly in CA-BSI isolates (9/24; 37.5% *versus* 4/41; 9.8%; *p* = 0.01). Two of the three carbapenem-resistant isolates carried the *bla*_KPC−like_ gene, while one had the *bla*_NDM−like_ gene. Although *bla*_TEM−like_, *bla*_SHV−like_, and *bla*_OXA−1−like_ genes were identified more frequently in CA-BSI isolates, the differences were not statistically significant.

**Table 3:** Patterns of β-lactamase genes in *Klebsiella pneumoniae* from community-acquired bloodstream infections (*n* = 24) and community-acquired urinary tract infections (*n* = 41).

β-lactamases genes patterns		Total of isolates	CA-BSI	CA-UTI	*p*
		*n* = 65 (%)	*n* = 24 (%)	*n* = 41 (%)	
A	*bla*_SHV−like_	40 (61.5)	11 (45.8)	29 (70.7)	0.07
B	*bla*_SHV−like_, *bla*_TEM−like_, *bla*_CTX−M−1_	4 (6.2)	3 (12.5)	1 (2.4)	0.14
C	*bla*_SHV−like_, *bla*_TEM−like_, *bla*_OXA−1−like_, *bla*_CTX−M−1_	4 (6.2)	1 (4.2)	3 (7.3)	> 0.99
D	*bla*_SHV−like_, *bla*_TEM−like_	3 (4.6)	1 (4.2)	2 (4.9)	> 0.99
E	*bla*_SHV−like_, *bla*_OXA−1−like_, *bla*_CTX−M−9_	1 (1.5)	1 (4.2)	–	0.37
F	*bla*_SHV−like_, *bla*_OXA−1−like_, *bla*_CTX−M−1_, *bla*_KPC−like_	1 (1.5)	1 (4.2)	–	0.37
G	*bla*_SHV−like_, *bla*_OXA−1−like_	1 (1.5)	1 (4.2)	–	0.37
H	*bla*_SHV−like_, *bla*_CTX−M−9_	1 (1.5)	1 (4.2)	–	0.37
I	*bla*_SHV−like_, *bla*_KPC−like_	1 (1.5)	1 (4.2)	–	0.37
J	*bla*_SHV−like_, *bla*_TEM−like_, *bla*_OXA−1−like_, *bla*_CTX−M−1_, *bla*_NDM−like_	1 (1.5)	1 (4.2)	–	0.37
K	None	8 (12.3)	2 (8.3)	6 (14.6)	0.70

BSI, bloodstream infection; CA, community-acquired; UTI, urinary tract infection.

### Virulence profile between CA-BSI and CA-UTI *K. pneumoniae* isolates

Fourteen distinct virulence patterns of *K. pneumoniae* (A to N) were identified (Table 4). The virulence profiles of the isolates were similar between the two groups studied, with almost all isolates (95.4%, 62/65) classified as c*Kp* (Table 5). Hv*Kp* strains were identified in one CA-BSI patient (4.2%, 1/24) and two CA-UTI patients (4.9%, 2/41). A wide variety of capsular serotypes were identified, including the relevant serotypes K1 in one CA-UTI isolate (2.4%, 1/41), K2 in one CA-BSI isolate (4.2%, 1/24) and in two CA-UTI isolates (4.9%, 2/41), and K54 in one CA-BSI isolate (4.2%, 1/24). Interestingly, one K2 strain was classified as c*Kp*. Other K or KL-types identified and *wzi* alleles (including six new alleles) are shown in Fig. 1.

**Table 4:** Patterns of virulence genes in *Klebsiella pneumoniae* from community-acquired bloodstream infections (*n* = 24) and community-acquired urinary tract infections (*n* = 41).

Virulence genes patterns		Total isolates	CA-BSI	CA-UTI	*p*
		*n* = 65 (%)	*n* = 24 (%)	*n* = 41 (%)	
A	*mrkD*,* entB*,* fimH*,* uge*,* wabG*,* ureA*	28 (43.1)	10 (41.7)	18 (43.9)	> 0.99
B	*mrkD*,* entB*,* fimH*,* uge*,* wabG*,* ureA*,* ybtS*	10 (15.4)	4 (16.7)	6 (14.6)	> 0.99
C	*mrkD*,* entB*,* fimH*,* uge*,* wabG*,* ureA*,* kfu*	8 (12.3)	3 (12.5)	5 (12.2)	> 0.99
D	*mrkD*,* entB*,* fimH*,* uge*,* wabG*,* ureA*,* ybtS*,* kfu*	6 (9.2)	3 (12.5)	3 (7.3)	0.66
E	*mrkD*,* entB*,* fimH*,* wabG*,* ureA*,* ybtS*	2 (3.1)	1 (4.2)	1 (2.4)	> 0.99
F	*mrkD*,* entB*,* fimH*,* wabG*,* ureA*	2 (3.1)	1 (4.2)	1 (2.4)	> 0.99
G	*mrkD*,* entB*,* fimH*,* wabG*,* ureA*,* kfu*	2 (3.1)	1 (4.2)	1 (2.4)	> 0.99
H	*mrkD*,* fimH*,* uge*,* wabG*,* ureA*,* kfu*,* allS*	1 (1.5)	–	1 (2.4)	> 0.99
I	*mrkD*,* entB*,* fimH*,* uge*,* wabG*,* ureA*,* ybtS*,* kfu*,* allS*,* iroB*,* iucA*, _*p*_*rmpA*, _*p*_*rmpA2*,* peg-344*	1 (1.5)	–	1 (2.4)	> 0.99
J	*entB*,* fimH*,* uge*,* wabG*,* ureA*	1 (1.5)	–	1 (2.4)	> 0.99
K	*entB*,* fimH*,* uge*,* wabG*,* ureA*,* kfu*	1 (1.5)	–	1 (2.4)	> 0.99
L	*entB*,* fimH*,* uge*,* wabG*,* ureA*,* kfu*,* allS*	1 (1.5)	–	1 (2.4)	> 0.99
M	*entB*,* fimH*,* uge*,* wabG*,* ureA*,* iroB*,* iucA*, _*p*_*rmpA*, _*p*_*rmpA2*,* peg-344*	1 (1.5)	–	1 (2.4)	> 0.99
N	*mrkD*,* entB*,* fimH*,* uge*,* wabG*,* ureA*,* iroB*,* iucA*, _*p*_*rmpA*,* peg-344*	1 (1.5)	1 (4.2)	–	> 0.99

BSI, bloodstream infection; CA, community-acquired; UTI, urinary tract infection.

**Table 5:** Characterization of the virulence profile of *Klebsiella pneumoniae* isolates from community-acquired bloodstream infections (*n* = 24) and community-acquired urinary tract infections (*n* = 41).

Virulence factors	Target genes	Overall	CA-BSI	CA-UTI	*p*
		65 (%)	24 (%)	41 (%)	
**Capsular serotype**					
K1	*magA*	1 (1.5)	–	1 (2.4)	> 0.99
K2	wzi_K2	3 (4.6)	1 (4.2)	2 (4.9)	> 0.99
K54	*wzi*_K54 ^a^	1 (1.5)	1 (4.2)	–	0.37
**Capsule-related factors**					
Regulator of mucoid phenotype A/A2	_p_*rmpA* ^b^	3 (4.6)	1 (4.2)	2 (4.9)	> 0.99
	_p_*rmpA2* ^b^	2 (3.1)	–	2 (4.9)	0.53
Biosynthesis of the outer core lipopolysaccharide	*wabG*	65 (100)	24 (100)	41 (100)	> 0.99
Biosynthesis of the capsule and smooth lipopolysaccharide	*uge*	59 (90.8)	21 (87.5)	38 (92.7)	0.66
**Siderophores**					
Enterobactin	*entB*	64 (98.5)	24 (100)	40 (97.6)	> 0.99
Yersiniabactin	*ybtS*	19 (29.2)	8 (33.3)	11 (26.8)	0.59
Aerobactin	*iucA* ^b^	3 (4.6)	1 (4.2)	2 (4.9)	> 0.99
Salmochelin	*iroB* ^b^	3 (4.6)	1 (4.2)	2 (4.9)	> 0.99
**Fimbriae**					
Adhesin type 1 fimbriae	*fimH*	65 (100)	24 (100)	41 (100)	> 0.99
Adhesin type 3 fimbriae	*mrkD*	61 (93.8)	24 (100)	37 (90.2)	0.29
***Klebsiella*** **iron uptake system**					
Iron transport and phosphotransferase function	*kfu*	20 (30.8)	7 (29.2)	13 (31.7)	> 0.99
**Allantoin metabolism**					
Utilization of allantoin as an alternative source of nitrogen and carbon	*allS*	3 (4.6)	–	3 (7.3)	0.29
**Urease**					
Utilization of urea as a nitrogen source	*ureA*	65 (100)	24 (100)	41 (100)	> 0.99
**Putative metabolite transporter**					
Gene located in the virulence plasmid	*peg-344* ^b^	3 (4.6)	1 (4.2)	2 (4.9)	> 0.99
**Serum resistance**					
Serum resistance-associated outer membrane lipoprotein	*traT*	–	–	–	–
**Alpha hemolysin**					
Toxin	*hlyA*	–	–	–	–
**Cytotoxic necrotizing factor-1**					
Toxin	*cnf-1*	–	–	–	–
**c** ***Kp***		62 (95.4)	23 (95.8)	39 (95.1)	> 0.99
**hv** ***Kp***		3 (4.6)	1 (4.2)	2 (4.9)	

BSI, bloodstream infection; CA, community-acquired; c*Kp*, classical *Klebsiella pneumoniae*; hv*Kp*, hypervirulent *Klebsiella pneumoniae;* UTI, urinary tract infections; ^a^ Identified by Sanger sequencing of the *wzi* gene; ^b^ Genes used as markers to differentiate c*Kp* and hv*Kp*.

### Comparative of molecular typing

To assess the clonal relationship among *K. pneumoniae* isolates, we conducted PFGE and MLST analyses (Fig. 1). PFGE results demonstrated remarkable clonal diversity, revealing 49 pulsotypes (designated A to AW). Six small clusters (B, D, E, M, Z, and AE, with ≥ 80% similarity) comprising two or three isolates, grouped isolates from CA-BSI and CA-UTI. In the MLST analysis, which included 59 isolates, 43 distinct STs were identified, further confirming the high clonal diversity observed. Notably, ST11 (6/59, 10.2%) and ST15 (5/59, 8.5%) were the most prevalent, and ST6852 was described for the first time in this study.

**Fig. 4:**
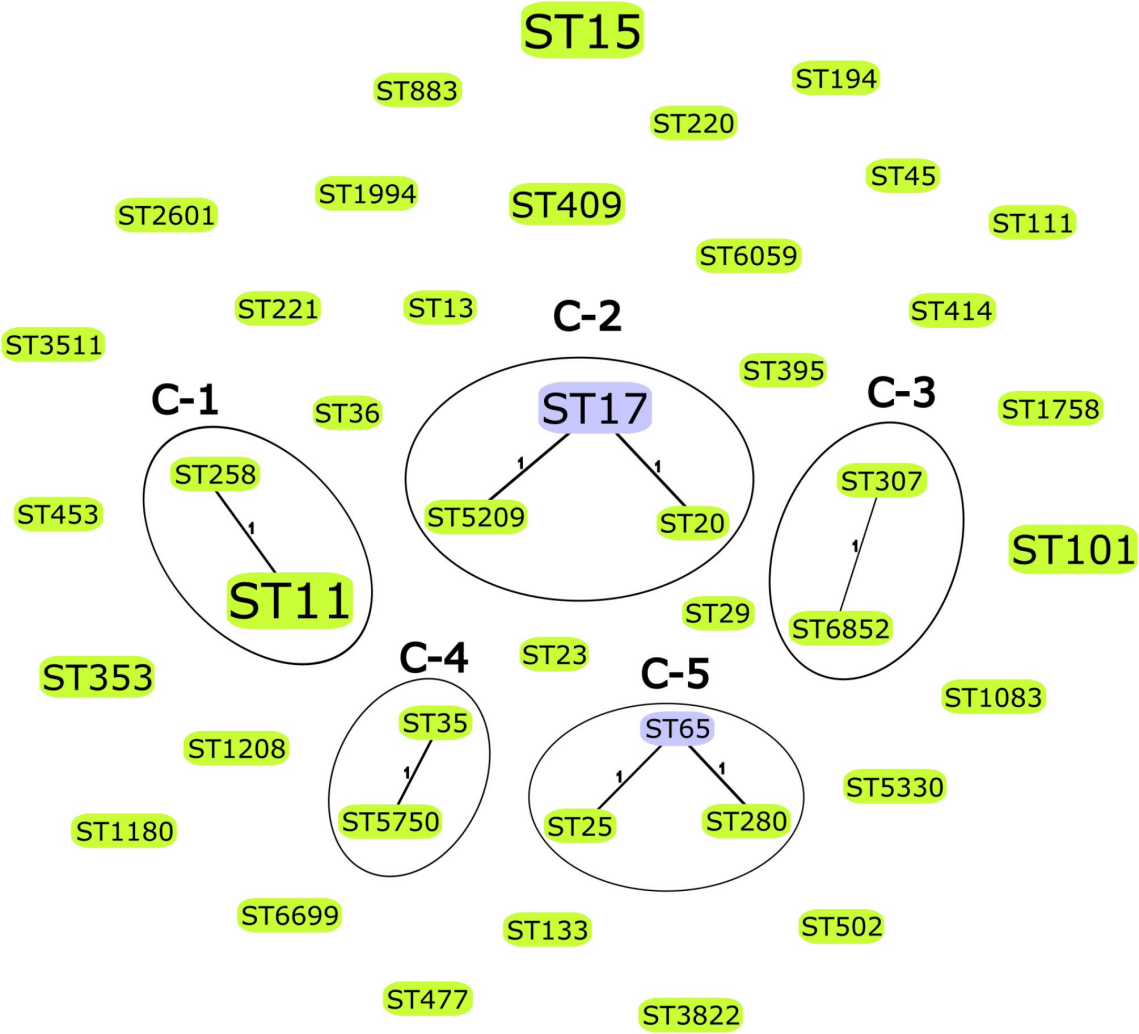
goeBURST-based population structure of 43 STs from 59 *Klebsiella pneumoniae* isolates. Five distinct clusters (C-1 to C-5) were identified, while 31 STs were singletons. The analysis displays only single-locus variants in a population snapshot. Connections between STs are represented by black lines, and the size of each node is proportional to the number of isolates corresponding to that ST in this study. Group founders are shown in light green, and common nodes are shown in blue. Legend: C, cluster; ST, sequence type.

The goeBURST analysis, considering only SLVs, revealed five distinct clusters. Cluster 1 (C-1) was composed of two STs (ST11 and ST258), accounting for 7/59 (11.9%) isolates. Cluster 2 (C-2) was formed by three STs: ST17, ST20, and ST5209, representing 6/59 (10.2%) isolates. Cluster 3 (C-3) consisted of ST307 and ST6852, comprising 2/59 (3.4%) isolates. Cluster 4 (C-4) included ST35 and ST5750, totaling 2/59 (3.4%) isolates, and Cluster 5 (C-5) was formed by ST25, ST65, and ST280, with 3/59 (5.1%) isolates. The remaining 31 STs were singletons and did not form any related clusters (Fig. 4). Regarding MDR isolates, these included ST11, ST15, ST101, ST194, ST258, ST280, ST307, ST395, and ST6852, while the hv*Kp* strains were represented by ST23, ST65, and ST5750.

## Discussion

*K. pneumoniae* is an important pathogen that causes BSI and UTI worldwide^1,32^. Although most studies focus on nosocomial or healthcare-associated infections, *K. pneumoniae* is frequently reported in CA infections^7,8,33–35^. This study revealed clinical and microbiological characteristics distinctions between patients with CA-BSI caused by *K. pneumoniae* and those with CA-UTI. These differences encompassed age, gender, comorbidities, and antimicrobial resistance patterns.

In line with prior research, most CA-BSI cases caused by *K. pneumoniae* occurred in elderly male patients with underlying conditions^6,9,33,36,37^. Host factors may partially explain these findings, as aging leads to a decline in immune fitness, the emergence of comorbidities, and alterations in the gut microbiome, thereby increasing both the frequency and severity of infectious diseases^38^. In this study, the urinary tract was the most common source of CA-BSI, aligning with findings from other studies^9,33^. Regarding studies on CA-UTI, most are biased towards including outpatients, resulting in limited studies for direct comparison. The only two studies with true cases we found also revealed a predominance of females; however, contrary to our findings, the patients were elderly (> 70 years), perhaps due to the inclusion of patients who needed to be hospitalized^7,8^. Like most studies on CA-BSI caused by *K. pneumoniae*, diabetes mellitus was identified as the most common underlying condition^6,9,13,33,34,36^. However, detailed information on the clinical conditions of patients with CA-UTI caused by *K. pneumoniae* is lacking in most studies, even in outpatients^5,14,16^. Nevertheless, Rozenkiewicz et al.^8^ also identified diabetes mellitus as the most prevalent underlying condition in patients with CA-UTI.

Kidney disease was more common in CA-BSI patients (20.8%) compared to previously reported literature^6,9,12,15,33–36^, with the exception of one study in New York (37.7%)^39^ and another in Taiwan (40.0%)^37^. Furthermore, the crude mortality observed in CA-BSI patients in our study was deemed high compared to findings in other studies^6,9,12,15,33,34,37^. The high prevalence of MDR *K. pneumoniae* strains among CA-BSI patients, particularly those who died (66.7%), may explain the elevated mortality rate observed in our study.

Most of the MDR *K. pneumoniae* isolates were obtained from elderly patients (87.5%), suggesting that the intestinal microbiota may serve as a potential reservoir. Older individuals are particularly susceptible to colonization by resistant bacteria due to physiological changes, prior antimicrobial exposure, and comorbidities^40^. Additionally, the high frequency of CA-BSI caused by MDR isolates secondary to UTI further supports this hypothesis.

Beyond the intestinal microbiota, horizontal gene transfer via plasmids and transposons likely plays a key role in the dissemination of β-lactamase genes within the community^41^. Previous studies have shown that ESBL and carbapenemase genes can be transmitted through direct contact, contaminated food, or healthcare-associated exposure, even in non-hospitalized individuals^42^. Further molecular epidemiological studies are needed to assess the genetic relatedness of these isolates and to better understand the mechanisms driving their resistance.

We observed that *K. pneumoniae* isolates from patients with CA-BSI exhibited higher resistance to antimicrobial agents than isolates from CA-UTI. However, we did not find studies with a similar methodological design for direct comparison. Upon reviewing previous studies individually, the prevalence of CA-BSI caused by ESBL-producing or carbapenem-resistant *K. pneumoniae* ranged from 0% up to 4% and 0% up to 0.4%, respectively, in investigations conducted in different countries^6,9,12,15,33–35,37,39^. Notably, the prevalences reported in the literature were lower than those in our study, where we identified 37.5% of ESBL producers and 12.5% of carbapenem resistance. It is important to note that infections caused by ESBL-producing or carbapenem-resistant bacteria result in significant morbidity and mortality worldwide^43^.

The prevalence of ESBL-producing *K. pneumoniae* in studies of CA-UTI varied, with 3.9% reported in Denmark^7^ and 35.3% in Spain^8^. These studies did not include data on the isolates’ susceptibility to carbapenems. In studies of *K. pneumoniae* isolates from UTI outpatients, the prevalence of ESBL production and carbapenem resistance was 12% and 0% in Portugal^14^ and approximately 32% and 17% in India^4^.

All ESBL-producing *K. pneumoniae* isolates in this study harbored *bla*_CTX−M−1_, *bla*_CTX−M−9_ or *bla*_KPC−like_ genes, which was more prevalent in isolates from patients with CA-BSI. Previous investigations of CA-BSI caused by *K. pneumoniae* were limited as they did not investigate resistance determinants. However, the *bla*_CTX−M_ gene has been the most frequently reported ESBL determinant in *K. pneumoniae* obtained from BSI^11,44^. Rozenkiewicz et al.^8^ and Richelsen et al.^7^ also did not investigate resistance genes in CA-UTI isolates. Nevertheless, Devi et al.^4^ reported *bla*_CTX−M_ as the most common ESBL determinant in UTI outpatients, while Caneiras et al.^14^ found other β-lactamases responsible for this phenotype.

The carbapenemase genes we identified were exclusively detected in isolates from CA-BSI, with *bla*_KPC−like_ identified in 8.3% and *bla*_NDM−like_ in 4.2% of the isolates. In UTI outpatients, carbapenemase genes were not found in a study conducted in Portugal^14^; however, *bla*_KPC_ was identified in 37.5% of isolates in a Brazilian study which did not specify whether the infections were CA or healthcare-related^5^. The detection of carbapenemase-producing *K. pneumoniae* in the community, particularly isolates carrying the *bla*_NDM_ gene, is a serious concern. It is important to note that no β-lactams or β-lactamase inhibitors combinations are currently available for clinical use against infections due to NDM-producing bacterial isolates^41^.

The virulence profile of *K. pneumoniae* isolates was similar between the two groups. However, one CA-BSI isolate (4.2%) and two CA-UTI isolates (4.9%) exhibited hv*Kp* biomarkers^29^. The hv*Kp* strain from the CA-BSI patient was identified as serotype K2, with the infection secondary to a liver abscess, a common infection site for these strains^2^. Notably, the prevalence of hv*Kp* strains in this study was lower than that reported in East Asian studies. For instance, Lee et al.^12^ and Juan et al.^37^ reported a prevalence of K1/K2 in approximately 35% of CA-BSI isolates, with hv*Kp* strains accounting for 59% of CA-BSI cases in a Taiwanese study^37^. Outside of Asia, a study in Canada detected *rmpA* in 8.2% of CA-BSI isolates^36^. Studies on UTI outpatients have investigated virulence factors, with a Taiwanese study reporting hv*Kp* in approximately 29% of cases^45^. No hv*Kp* biomarkers were detected in studies from Portugal^14^ and Brazil^5^.

Regarding molecular genotyping, both MLST and PFGE analyses revealed high clonal diversity. The few pulsotype clusters identified by PFGE generally corresponded to a single ST or its SLVs; however, discrepancies were observed in some groups with less than 90% similarity. These differences highlight the complementary nature of these techniques in characterizing clonal relationships. The majority of MDR isolates in this study (13/16, 81.3%) belonged to high-risk pandemic clonal groups (CG) in humans^46^: CG15 (ST15), CG101 (ST101), CG258 (ST258 and ST11), and CG307 (ST307 and ST6852). Although *K. pneumoniae* displays a diverse population structure, a small number of high-risk MDR clones account for more than half of the genomes in global collections^47^. This pattern reinforces the role of epidemic clones in the dissemination of antimicrobial resistance. Notably, ST6852, a SLV of ST307, was identified for the first time in this study.

High-risk hv*Kp* pandemic clones such as ST23 and ST65, which we identified in this study, have previously been reported in strains obtained from BSI^48,49^. The ST23 clone is frequently associated with liver abscesses, as evidenced by a reported case in Brazil^50^, and another Brazilian study reported this clone in polymyxin-resistant *K. pneumoniae* carrying *bla*_KPC_ and other resistance genes^51^. Additionally, ST5750 was reported for the first time in a hv*Kp* isolate causing human infection in Guadeloupe, France^52^.

The major limitation of this study was the relatively small sample size of CA-BSI patients, which restricted the statistical power of the analysis. However, true CA-BSI cases are uncommon, and a careful evaluation of the patient’s clinical history is necessary to avoid including patients with healthcare-acquired infections. Additionally, the identification of *bla*_SHV−like_ and *bla*_TEM−like_ genes without variant specification limited a more detailed understanding of the resistance mechanisms involved, as certain variants are known to be associated with ESBL production.

In conclusion, we have demonstrated that CA-BSI caused by *K. pneumoniae* differs from CA-UTI in clinical and microbiological characteristics. Older age, male sex, more comorbidities, and an increased proportion of multidrug-resistant isolates—particularly ESBL-producing and carbapenem-resistant strains—were significantly associated with CA-BSI cases. Notably, the proportion of resistant *K. pneumoniae* isolates in community-acquired infections in Brazil was higher than those reported in other regions, raising concerns about the spread of high-risk pandemic clones. These findings underscore the urgent need for enhanced surveillance and preventive measures, particularly in Brazil, where a comprehensive resistance monitoring system for community-acquired infections is lacking.

## Electronic supplementary material

Below is the link to the electronic supplementary material.

Supplementary Material 1

## Data Availability

The data supporting the findings of this study are included in the manuscript or supplementary information. The sequences of the new *wzi* alleles identified in this study have been deposited in the BIGSdb-Pasteur *Klebsiella* database (https://bigsdb.pasteur.fr/klebsiella/) under accession numbers 742, 743, 744, 745, 746, and 747. All other alleles identified were identical to previously reported sequences, also available in the same database.
