# Supplementary material for: High proportions of multidrug-resistant Klebsiella pneumoniae isolates in community-acquired infections, Brazil
Source: Sci Rep. 2025 Mar 20;15:9698. doi: 10.1038/s41598-025-92549-w (PMC11926192; doi:10.1038/s41598-025-92549-w)
Supplement: Supplementary file 1 — Supplementary Material 1 [file 41598_2025_92549_MOESM1_ESM.docx]

**Supplementary File**

**Scientific Reports**

**High proportions of multidrug-resistant *Klebsiella pneumoniae* isolates in community-acquired infections, Brazil**

Adriano de Souza Santos Monteiro^1^, Marcio de Oliveira Silva^2^, Maria Goreth Barberino^2^, Lorena Galvão de Araujo^1^, Edilane Lins Gouveia^3^, Adriele Pinheiro Bomfim^4^, Camila Maria Piñeiro Silva^5^, Vívian Santos Galvão^4,5^, Soraia Machado Cordeiro^5^, Joice Neves Reis^1,4,5^*

**Affiliation(s)**

^1^ Postgraduate Course in Biotechnology in Health and Investigative Medicine, Gonçalo Moniz Institute, Oswaldo Cruz Foundation, Salvador, Bahia, Brazil

^2^ São Rafael Hospital, Salvador, Bahia, Brazil

^3^ Bahia Hospital, Salvador, Bahia, Brazil

^4^ Postgraduate Program in Pharmacy, Faculty of Pharmacy, Federal University of Bahia, Salvador, Bahia, Brazil

^5^ Faculty of Pharmacy, Federal University of Bahia, Salvador, Bahia, Brazil

***Corresponding author**: Joice Neves Reis, School of Pharmacy, Federal University of Bahia, Ondina, 40170-115, Salvador, Bahia, Brazil. **E-mail: joice@ufba.br**

**Table S1** List of primers and product sizes of β-lactamase, capsular type, and virulence genes investigated in this study

| **β-lactamase genes** | **Primers** | **Base pair** | **Reference** |
| --- | --- | --- | --- |
| *bla*_SHV-like_ | F: AGCCGCTTGAGCAAATTAAAC | 713 | 26 |
|  | R: ATCCCGCAGATAAATCACCAC |  |  |
| *bla*_TEM-like_ | F: CATTTCCGTGTCGCCCTTATTC | 800 | 26 |
|  | R: CGTTCATCCATAGTTGCCTGAC |  |  |
| *bla*_OXA-1-like_ | F: GGCACCGATTCAACTTTCAAG | 564 | 26 |
|  | R: GACCCCAAGTTTCCTGTAAGTG |  |  |
| *bla*_CTX-M-1_ | F: TTAGGAARTGTGCCGCTGYA | 688 | 26 |
|  | R: CGATATCGTTGGTGGTRCCAT |  |  |
| *bla*_CTX-M-2_ | F: CGTTAACGGCACGATGAC | 404 | 26 |
|  | R: CGATATCGTTGGTGGTRCCAT |  |  |
| *bla*_CTX-M-9_ | F: TCAAGCCTGCCGATCTGGT | 561 | 26 |
|  | R: TGATTCTCGCCGCTGAAG |  |  |
| *bla*_GES-like_ | F: AGTCGGCTAGACCGGAAAG | 399 | 26 |
|  | R: TTTGTCCGTGCTCAGGAT |  |  |
| *bla*_OXA-48-like_ | F: GCTTGATCGCCCTCGATT | 281 | 26 |
|  | R: TTCGGCTTGACTCGGCTGA |  |  |
| *bla*_IMP-like_ | F: TTGACACTCCATTTACDG | 139 | 26 |
|  | R: GATYGAGAATTAAGCCACYCT |  |  |
| *bla*_VIM-like_ | F: GATGGTGTTTGGTCGCATA | 390 | 26 |
|  | R: CGAATGCGCAGCACCAG |  |  |
| *bla*_KPC-like_ | F: CATTCAAGGGCTTTCTTGCTGC | 538 | 26 |
|  | R: ACGACGGCATAGTCATTTGC |  |  |
| *bla*_NDM-like_ | F: TCCTTGATCAGGCAGCCACC | 591 | 25 |
|  | R: CGCATTAGCCGCTGCATTGA |  |  |
|  |  |  |  |
| **Capsular type genes** |  |  |  |
| K1 | F: GGTGCTCTTTACATCATTGC | 1283 | 27 |
|  | R: GCAATGGCCATTTGCGTTAG |  |  |
| K2 | F: CAACCATGGTGGTCGATTAG | 531 | 27 |
|  | R: TGGTAGCCATATCCCTTTGG |  |  |
| *wzi* | F: GTGCCGCGAGCGCTTTCTATCTTGGTATTCC | 580 | 31 |
|  | R: GAGAGCCACTGGTTCCAGAA(C/T)TT(C/G)ACCGC |  |  |
|  |  |  |  |
| **Virulence genes** |  |  |  |
| *_p_rmpA* | F: GAGTAGTTAATAAATCAATAGCAAT | 332 | 29 |
|  | R: CAGTAGGCATTGCAGCA |  |  |
| *_p_rmpA_2_* | F: GTGCAATAAGGATGTTACATTA | 430 | 29 |
|  | R: GGATGCCCTCCTCCTG |  |  |
| *wabG* | F: CGGACTGGCAGATCCATATC | 683 | 30 |
|  | R: ACCATCGGCCATTTGATAGA |  |  |
| *uge* | F: GATCATCCGGTCTCCCTGTA | 534 | 30 |
|  | R: TCTTCACGCCTTCCTTCACT |  |  |
| *entB* | F: GTCAACTGGGCCTTTGAGCCGTC | 400 | 27 |
|  | R: TATGGGCGTAAACGCCGGTGAT |  |  |
| *ybtS* | F: GACGGAAACAGCACGGTAAA | 242 | 27 |
|  | R: GAGCATAATAAGGCGAAAGA |  |  |
| *iucA* | F: GCTTATTTCTCCCCAACCC | 583 | 29 |
|  | R: TCAGCCCTTTAGCGACAAG |  |  |
| *iroB* | F: ATCTCATCATCTACCCTCCGCTC | 235 | 29 |
|  | R: GGTTCGCCGTCGTTTTCAA |  |  |
| *fimH* | F: ATGAACGCCTGGTCCTTTGC | 688 | 28 |
|  | R: GCTGAACGCCTATCCCCTGC |  |  |
| *mrkD* | F: AAGCTATCGCTGTACTTCCGGCA | 340 | 27 |
|  | R: GGCGTTGGCGCTCAGATAGG |  |  |
| *kfu* | F: GGCCTTTGTCCAGAGCTACG | 638 | 27 |
|  | R: GGGTCTGGCGCAGAGTATGC |  |  |
| *allS* | F: CATTACGCACCTTTGTCAGC | 764 | 27 |
|  | R: GAATGTGTCGGCGATCAGCTT |  |  |
| *ureA* | F: GCTGACTTAAGAGAACGTTATG | 337 | 30 |
|  | R: GATCATGGCGCTACCT(C/T)A |  |  |
| *traT* | F: GGTGTGGTGCGATGAGCACAG | 290 | 28 |
|  | R: CACGGTTCAGCCATCCCTGAG |  |  |
| *hlyA* | F: AACAAGGATAAGCACTGTTCTGGCT | 1177 | 28 |
|  | R: ACCATATAAGCGGTCATTCCCGTCA |  |  |
| *cnf*-1 | F: AAGATGGAGTTTCCTATGCAGGAG | 498 | 28 |
|  | R: CATTCAGAGTCCTGCCCTCATTATT |  |  |
| *peg-344* | F: AAAGGACAGAAAGCCAGTG | 411 | 29 |
|  | R: CAATGACGAGGGGGATAATC |  |  |
